# Effects of “accurate measurement” comprehensive sports activities on balance ability, body composition and bone density of female college students

**DOI:** 10.3389/fphys.2023.1117635

**Published:** 2023-05-09

**Authors:** Zhilei Zhang, Jie Liu, Jianguo Li, Jianping Li

**Affiliations:** Department of Physical Education and Health, Heze University, Heze, China

**Keywords:** balance ability, body composition, bone mineral density, exercise intervention, college girl

## Abstract

**Background:** A sedentary lifestyle with little movement has affected modern youth, and regular exercise has real benefits for people; such studies are mostly for older adults, and more evidence is needed for adolescents.

**Objective:** To compare differences in balance, body composition, and bone mineral density among female college students before and after an exercise intervention to provide precise evidence that exercise promotes college student health.

**Methods:** A whole group of female students in a university was sampled and included in the statistical analysis 50 people, divided into two cohorts, 21 people in the test group and 29 people in the control group; the test group had 4 comprehensive sports activities per week and the control group had 1 comprehensive sports activities per week, and the differences in each index of balance ability, body composition and bone density before and after the intervention were compared after 3 months.

**Results:** After exercise intervention, when maintaining balance, the area of the center of gravity movement trajectory increased by 32.36% in the test group compared with the pre-intervention period and increased by 42.80% compared with the control group, and the differences were all statistically significant (*p* < 0.01); body mass index (BMI), body fat rate (BFR), visceral fat area (VFA), skeletal muscle content, and Inbody score increased over time more reasonable, and the difference in the effect of time factor (effect) was statistically significant (*p* < 0.01); bone mineral density (BMD) and BMD Z value increased with time, and the difference in the effect of time factor was statistically significant (*p* < 0.05).

**Conclusion:** Female college students’ body balance ability improved substantially after exercise intervention; at the university level, female college students had a more rational body composition and continued natural increase in BMD, which were not related to exercise intervention.

## 1 Introduction

With the advancement of technology, people’s lifestyles have changed a lot, such as smartphone use, which increases sedentary time and reduces physical activity time, especially for young people (González-ruiz et al., 2018; Guthold et al., 2018; Kemp et al., 2022). Physical inactivity is the cause of many chronic diseases and musculoskeletal disorders, such as sarcopenia and osteoporosis, which place a significant health and social service burden on countries. The international community has recognized physical activity as a priority for health promotion and physical activity as an effective non-pharmaceutical intervention. Human balance is the sum of complex reflexes that coordinate the movement of all parts of the body based on proprioceptive, visual and vestibular sensory information through the regulation of the central nervous system (Jin and Yu, 2005). Balance plays an important role in daily life, not only as one of the main abilities for physical activity, but also as one of the main scientific bases for fall prevention. Body composition is an important indicator to accurately evaluate the health level and body size standard of adolescents, which can monitor their nutritional status and growth and development, and has an important impact on athletic ability; body composition in adolescence has strong plasticity and is a critical period for benign development. Bone mineral density (BMD), is an important indicator of bone health and is used to assess bone strength. The survey shows that the low bone mass rate of people over 50 years old in China is 46.4%, including 46.9% for men and 45.9% for women (Chinese Medical Association Division of Osteoporosis and Bone Mineral Salt Diseases, 2019).

Physical activity history in relation to body composition and functional capacity (Bland et al., 2021; Buckinx et al., 2021). Meta-analytical studies of resistance exercise are effective in improving lower limb muscle strength, increasing lean body mass and reducing body fat in cancer patients (Padilha et al., 2017). One study used 12 weeks of high-intensity interval training to improve physical function and body composition in sedentary obese older adults, with positive results (Buckinx et al., 2019). An analysis of the association between environmental factors and their bone mass in 200 young people showed that body mass index (BMI) and physical activity level were significantly associated with bone health in young people (Correa-rodriguez et al., 2018). Young female athletes participating in high weight-bearing activity sports exhibit higher levels of bone mineral density in the lower extremities (Helder et al., 2018). One study measured higher whole-body bone density in female runners than in female non-runners (Mccormack et al., 2019). 16-week comprehensive exercise training intervention for HIV-infected patients results in increased muscle strength and improved depression and quality of life indices compared to recreational group (Oliveira et al., 2020). Muscle atrophy is characterized by a reduction in muscle mass and strength, which can lead to a number of age-related diseases. The loss of muscle mass can directly affect the quality of daily life and is a major risk factor for chronic diseases (Brunetta et al., 2020). Physical activity is important for maintaining health by promoting muscle protein synthesis and activating signaling pathways that regulate muscle fiber metabolism and function (Shen et al., 2018). Positive correlation between BMD and skeletal muscle (ASM) mass of the extremities in 1,343 men aged 40 years or older in a Chinese community at three-year follow-up (Han et al., 2021). There is a significant positive correlation between muscle mass, bone density and adiposity in elderly women. Appropriate exercise and nutritional support can increase muscle mass, improve bone density and promote the positive effect of adipose tissue, which is conducive to improving balance, preventing the risk of falls, fractures and disability and improving the quality of life of the elderly (Yin et al., 2022). There is an investigation into the characteristics of changes in age, BMI and body composition in postmenopausal women and the relationship between them and osteoporosis. Age is a risk factor for osteoporosis in postmenopausal women, while BMI, muscle mass and fat mass are protective factors that contribute to overall BMD and overall bone mineral content (Yuan et al., 2022a). Analysis of clinical data from 1,032 cases of postmenopausal women in China showed that skeletal muscle index of the extremities was negatively associated with the risk of osteoporotic fracture at major sites and the risk of hip fracture (Lin et al., 2022a). Postmenopausal women’s bone mineral density is closely related to changes in skeletal muscle mass (Zhao et al., 2011). Women are more likely to suffer from osteoporosis, and body composition can be used as an indirect reference indicator to evaluate bone health status (Yuan et al., 2022b). Study finds positive health effects of team sports training for sedentary women (Møller et al., 2020).

In review, studies on the effects of exercise on physiological function, muscle and bone density have focused on areas related to promoting recovery from disease (Varahra et al., 2018; Schootemeijer et al., 2020; Chen et al., 2022; Mavropalias et al., 2022; Talotta et al., 2022), or for middle-aged and elderly populations (Du et al., 2022; Papp et al., 2022), or for postmenopausal women (Shojaa et al., 2020; Hejazi et al., 2022), studies on adolescents are relatively fewer. However, the health problems caused by sedentary behavior of college students cannot be ignored. Female college students are in late adolescent development, their body physique is gradually becoming adult and fixed, and their health status is of great significance for future work and childbirth. Before and after puberty is a critical stage of bone density development, and obtaining the highest possible bone mass plays an important role in preventing the occurrence of osteoporosis (Zhang et al., 2000). Late adolescence is the 2nd critical period for maintaining bone mass, lasting 2–5 years, during which appropriate physical training can help maintain peak bone mass (Li et al., 2012). Physical exercise strengthens the muscles and bones as a whole unit and can improve muscle strength and body balance. There is a correlation between muscle activity and bone density (Calendo et al., 2014; Cidem et al., 2014). Muscles and bones are closely related, the two complement each other, scientific exercise will play a dual effect (Zhao et al., 2017). At this stage, the myopia rate of Chinese school students is rapidly rising and overweight is increasing year by year. Some scholars suggest that “accurate measurement”, “accurate analysis” and “accurate intervention” of students’ physical fitness is the key to solve the development of students’ physical health (This publication reports, 2020). Therefore, to conduct exercise intervention for female college students, professional intervention personnel, precise instruments, and specialized testers were used to accurately measure three aspects of balance ability, body composition, and BMD, and to compare the differences of each index before and after the intervention. School physical education classes generally include a variety of physical exercises to improve overall physical fitness, and this study selected integrated physical activities that included running, jumping, and throwing for the intervention. It is hypothesized that comprehensive sports activities has a good effect on balance ability, body composition, and BMD, providing more evidence for female college students’ exercise for health.

## 2 Materials and methods

### 2.1 Subjects and exercise intervention

**FIGURE 1:**
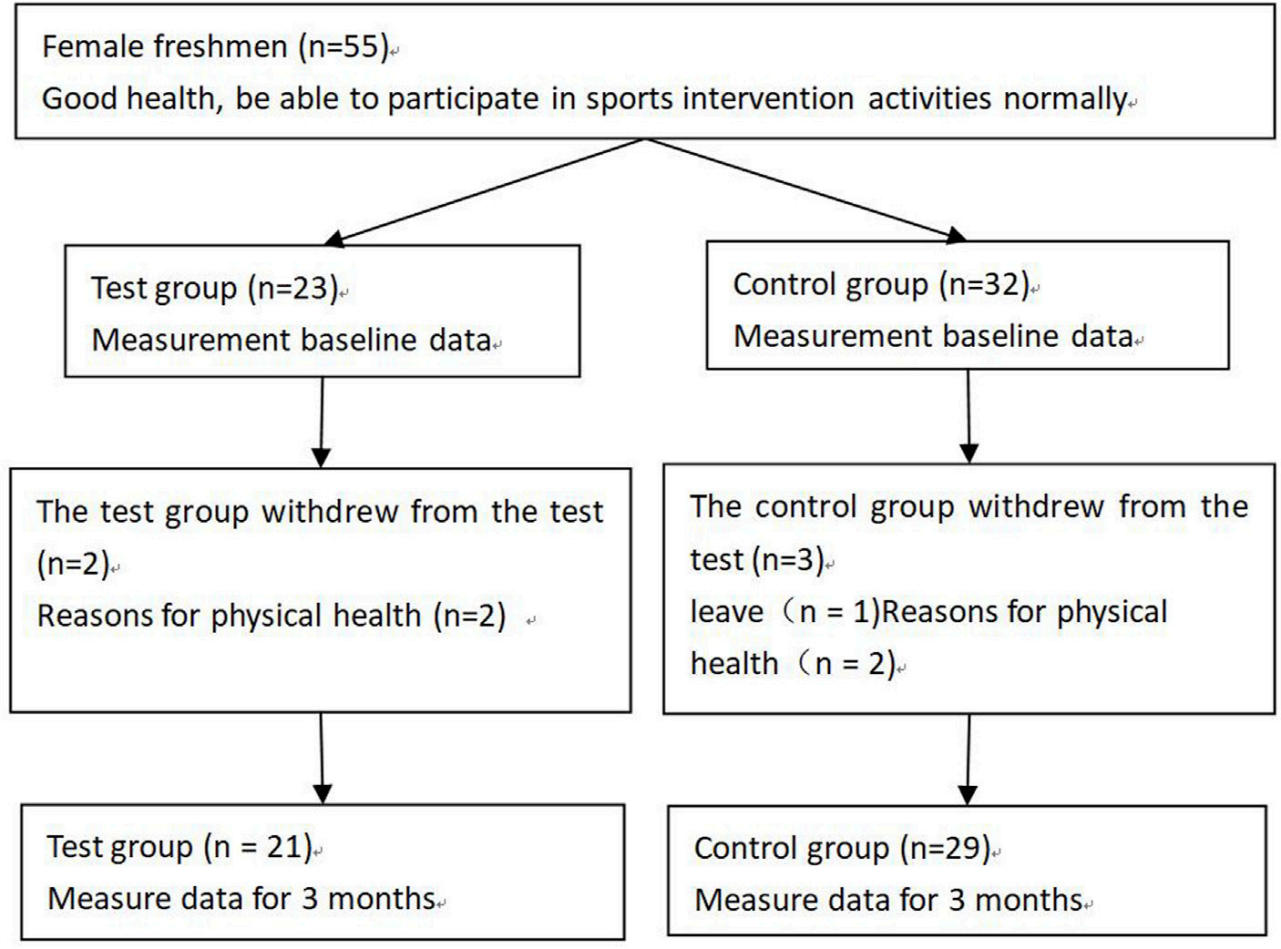
Flowchart of intervention process.

The subjects were female college students, and the sample size was estimated by G*Power3.1.7. Inclusion Criteria: Healthy and normal participation in sports intervention activities. Exclusion criteria: unable to complete physical activities as planned and unable to participate in the test on time. A whole cohort sample of female students in two first-year university classes at a general university, with 23 in the test group (class) and 32 in the control group (class), for a total of 55 students. The intervention was organized 4 times a week for 100 min of comprehensive sports activities in the test group and 1 time a week for 100 min of comprehensive sports activities in the control group, with a 3-month follow-up. Comprehensive sports activities include running, jumping, throwing, coordination and flexibility exercises with jogging, stretching, aerobics, basketball activities, standing long jump activities, *etc.* The intervention was implemented by university physical education teachers at school teaching sites with 90% or more attendance. The intervention flow chart is shown in Figure 1. All subjects signed a written informed consent form. The study was approved by the Academic Ethics Committee of Heze University. The study was registered with the Chinese Clinical Trials Registry under the number ChiCTR2000039041.

### 2.2 Tools and data acquisition

**FIGURE 2:**
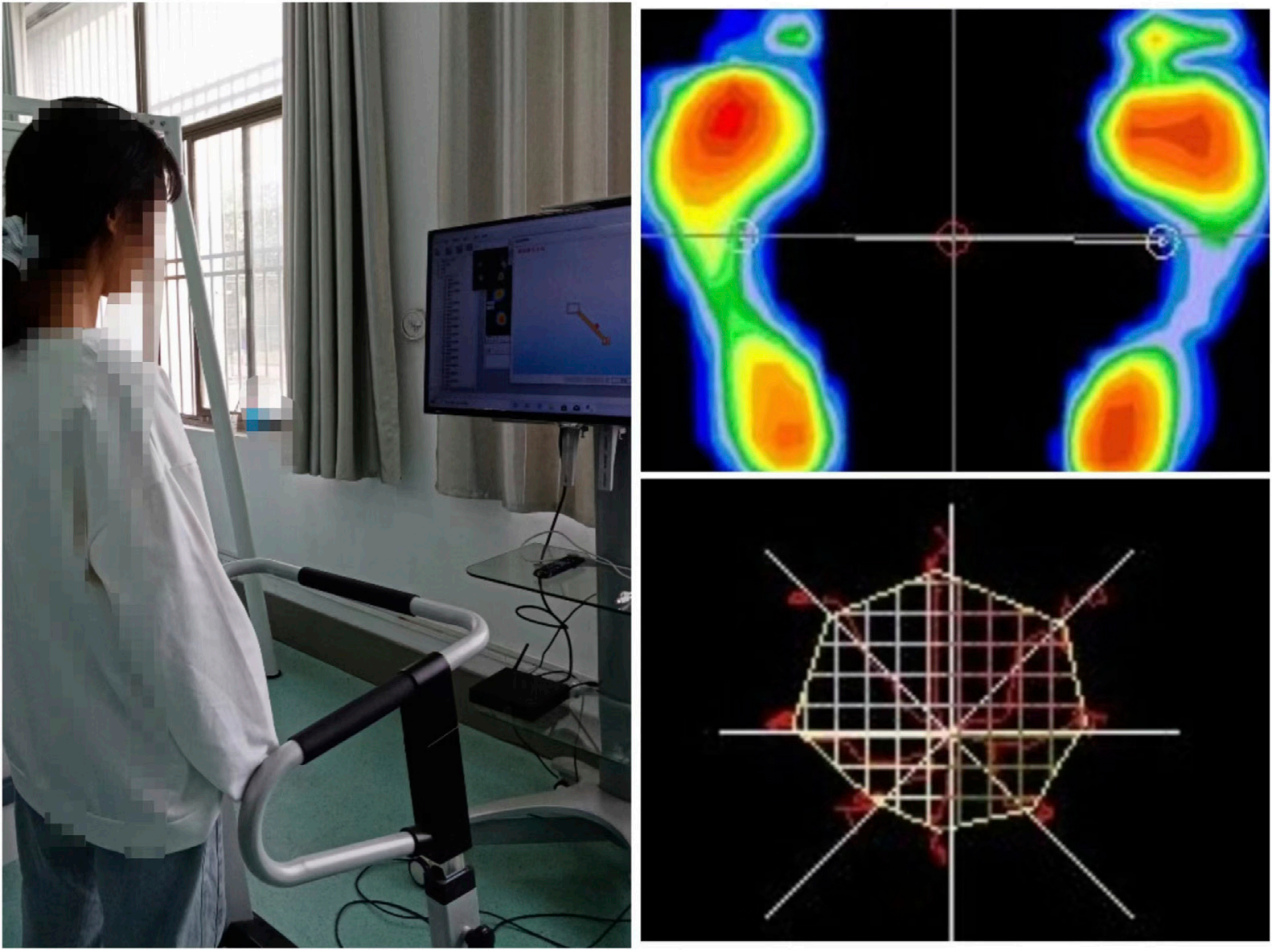
Balance test.

The balance ability test was performed using the Balance Function Training and Assessment System (Model: XY-PH-111, China). The subjects stood barefoot on the test platform, calibrated the footprint position according to the display in front of them, made the center of gravity cast point at the cross center marked on the screen, and tilted their bodies to the limit of stability in 8 directions: forward, right front, right, right back, back, left back, left and left front according to the screen arrow prompts. The subject observes his or her center of gravity on the display and tilts as far as possible in the direction of maximum tilt for more than 2 s. During the whole test, if the subject’s feet move or fall, the test is repeated. The tester is mainly composed of three parts: pressure sensor (test platform), computer and application software. The pressure sensor can record the swaying of the body’s center of gravity, and convert the recorded signal into data input to the computer, the computer, with the support of application software, analyzes the received data, describes the projection trajectory of the body’s center of gravity on the pressure plate in real time, and automatically records the trajectory of the body’s center of gravity movement, the area formed by the trajectory (balance area) can record a very small amount of center of gravity movement, which can be compared quantitatively The area formed by the trajectory (balance area) can be recorded to a very small amount of center of gravity movement, which can reflect the balance function in a more quantitative and objective way and facilitate comparison between different testers. See Figure 2.

Body composition testing was performed using the Biospace body composition analyzer (model: InBody 770, South Korea). Using bioelectrical impedance analysis (BIA), a non-invasive method that involves placing electrodes on the feet and hands, low-frequency electric current is passed through the entire body, and the flow of current will be affected by body water. The BIA device measures how the current signal is obstructed by different types of tissue (muscle has high conductivity, but fat can slow down the signal), and after the BIA determines the resistance to the flow of current through the body, body fat and other body composition data can be calculated using an equation based on the value of body water. Body composition was selected from five indicators that are closely related to the physical health of female university students. Body Mass Index (BMI), BMI (kg/m^2^) is a standard commonly used internationally to measure the degree of body fat and thinness and whether it is healthy or not, and the calculation formula is: BMI = weight ÷ height^2^; Body Fat Rate (BFR) is the proportion of body fat weight in the total body weight (%), which reflects the amount of fat content in the human body. Visceral fat area (VFA) is the area of visceral fat (cm^2^) in CT image of abdominal belly button section, which is one of the test indicators of body composition analyzer and is an important indicator to evaluate whether it is hidden obesity. Skeletal muscle content (kg) directly reflects the body’s activity function, and any activity is the result of skeletal muscle contraction. The Inbody score is based on the difference between muscle mass and fat mass measurements and the standard value. The base score is 80 points, and the muscle mass measurement is lower than the standard value.

Bone density testing was performed using an OsteoSys dual-energy X-ray bone densitometer (model: EXA-3000, South Korea). The left ulnar flexure (forearm) distal bone mineral density (BMD) and Z value were tested; Z value indicates the ratio of the subject’s BMD to the average peak BMD of the same age, the same sex, and the same race, and the Z value can be used to understand the position of the subject’s BMD compared to that of the same age, indicating a higher or lower BMD value than that of people of the same sex, age, and race. The testers are specially trained and the tests are conducted in specialized laboratories.

### 2.3 Statistical analysis

The data were imported into SPSS20.0 statistical software. Descriptive statistics were used for the basic situation of the subjects, and independent sample *t*-test was used for comparison between different groups of baselines. When examining the effect of exercise on the balance ability, body composition and bone mineral density of female college students, the repeated measurement analysis of variance of 2 (test time: pre-test, post-test) × 2 (group: test group, control group) was used to compare the interaction within the group (pre-test, post-test) and between groups. The spherical test was carried out before the repeated measurement analysis of variance, and the Greenhouse-Geisser method was used when the football shape was not satisfied. If the interaction was significant, the Bonferroni correction was used for post-test, and the intra-group and inter-group differences were compared through simple effect analysis. In the statistical analysis, in order to reflect the test effect, the effect quantity index η^2^ is reported, which is considered to be a small effect when η^2^ = 0.01, a medium effect when η^2^ = 0.06, and a large effect when η^2^ = 0.14. When *p* < 0.05, the difference was statistically significant.

## 3 Results

### 3.1 Basic information of the subjects

A total of 50 female college students were included in the statistical analysis, including 21 in the test group and 29 in the control group (Table 1). It is considered that the baseline of the test group and the control group is balanced.

**TABLE 1:** Comparison of basic conditions between the test group and the control group (*n* = 50,‾x ± S).

Subjects	Test group (*n* = 21)	Control group (*n* = 29)	*t*	*P*
age (year)	19.48 ± 0.81	19.24 ± 0.64	1.15	0.26
height (cm)	163.47 ± 4.80	162.79 ± 7.20	0.37	0.71
weight (kg)	58.11 ± 8.70	59.74 ± 12.17	−0.52	0.60

### 3.2 The influence of exercise on the balance ability of female college students

The differences were not statistically significant in the independent sample *t*-test between the test and control groups for the premeasured balance area. (*t* = 0.62, *p* = 0.54). A 2 × 2 repeated-measures ANOVA was used to determine the difference in the change of human balance area over time between the test and control groups (Table 2). Because the interaction between group and time had a statistically significant effect on the balance area (*p* < 0.0L), the simple effect analysis of the two factor groups and time showed that the post-test in the test group increased 2814 mm^2^ by 32.36%, and the difference was statistically significant (*p* < 0.0L). The post-test in the control group decreased 128 mm^2^ compared with the pre-test, but the difference was not statistically significant (*p* = 0.84); In the post-test, the test group had more 3450 mm^2^ than the control group, an increase of 42.80%, and the difference was statistically significant (*p* < 0.01).

**TABLE 2:** Comparison of balance area between the two groups of college students before and after the experiment (*n* = 50,‾x ± S).

	Test group (*n* = 21)		Control group (*n* = 29)		Time *F* value (η^2^)	Time × group *F* value (η^2^)
	Pretest	Posttest	Pretest	Posttest		
balance area (mm^2^)	8,696 ± 2,435	11,510 ± 3,666	8,188 ± 3,153	8,060 ± 2,729	8.06 (0.14)*	9.68 (0.17)**

Note: *p < 0.05, **p < 0.01.

### 3.3 The influence of exercise on the body composition of female college students

Body composition is closely related to body mass index (BMI), body fat rate (BFR), visceral fat area (VFA), skeletal muscle content and Inbody score. There was no significant difference in BMI, BFR, VFA, skeletal muscle content and Inbody score between the test group and the control group (*p* > 0.05). A 2 × 2 repeated-measures ANOVA was used to determine the differences in the changes of each index between the test and control groups over time (Table 3).

**TABLE 3:** Comparison of body composition indicators between the two groups of college students before and after the experiment (*n* = 50,‾x ± S).

	Test group (*n* = 21)		Control group (*n* = 29)		Time *F* value (η^2^)	Time×group *F* value (η^2^)
	Pretest	Posttest	Pretest	Posttest		
BMI(kg/m^2^)	21.74 ± 2.75	21.39 ± 2.52	22.35 ± 3.23	22.06 ± 3.48	9.80 (0.17)**	0.07 (<0.01)
BFR(%)	31.94 ± 5.92	29.73 ± 5.67	33.81 ± 4.14	30.99 ± 4.83	87.77 (0.65)**	1.31 (0.03)
VFA (cm^2^)	87.35 ± 30.53	76.78 ± 26.52	99.01 ± 34.83	86.04 ± 35.79	59.19 (0.55)**	0.61 (0.01)
Skeletal muscle (kg)	21.03 ± 2.70	21.39 ± 2.43	21.03 ± 3.60	21.61 ± 3.68	24.02 (0.33)**	1.49 (0.03)
Inbody score	69.29 ± 5.07	71.48 ± 4.84	68.45 ± 3.28	71.34 ± 3.60	80.51 (0.63)**	1.55 (0.03)

Note: **p < 0.01.

### 3.4 Effect of exercise on bone mineral density of female college students

There was no significant difference in bone mineral density (BMD) and BMD Z value between the test group and the control group (*p* > 0.05). A 2 × 2 repeated-measures ANOVA was used to determine the differences in the change of each index between the test and control groups over time (Table 4).

**TABLE 4:** Comparison of bone mineral density between the two groups of college students before and after the experiment (*n* = 50,‾x ± S).

	Test group (*n* = 21)		Control group (*n* = 29)		Time *F* value (η^2^)	Time × group *F* value (η^2^)
	Pretest	Posttest		Pretest		
BMD(g/cm^3^)	0.43 ± 0.07	0.44 ± 0.07	0.41 ± 0.05	0.44 ± 0.06	6 (0.11)*	0.20 (<0.01)
Z (g/cm^3^)	−0.79 ± 1.01	−0.51 ± 1.24	−1.05 ± 0.72	−0.64 ± 0.94	5.64 (0.11)*	0.19 (<0.01)

Note: *p < 0.05.

## 4 Discussion

The results of the study female college students after exercise intervention, the balance area of the test group increased substantially, and the control group decreased slightly, proving that exercise is important for enhancing the balance ability of female college students. Studies have demonstrated that exercise is beneficial in improving functional capacity and dynamic balance in children with hemiplegia (Soliman et al., 2022), a randomized controlled trial showed that a gait training program used by stroke patients was beneficial in improving balance (Shaw et al., 2022), and another study female university students through 8 weeks of fitness qigong exercises. The result was a significant increase in balance test scores post-intervention compared to pre-intervention scores (Su et al., 2018). These are similar to the results of this study, and the fact that healthy female college students can consistently improve their balance through comprehensive sports activitiesis an exciting and noteworthy result. Balance is an important physiological function of the human body, which is the ability to maintain the posture of the body; especially the ability to control the body’s center of gravity on a small support surface. Balance is an important safeguard for the body to maintain standing, walking and coordinating various movements. The postural balance of the human body depends on the coordination of visual, proprioceptive and vestibular information and the control of motor effectors by the central system. Information from the visual system is collected by the retina and transmitted via the visual pathway to the visual center, providing information about the surrounding environment and the body’s movement and direction; proprioception transmits information about the state of muscles, joints, tendons and other effector organs; and vestibular sensation is the main structure for maintaining balance and perceiving the body in relation to the surrounding environment. The main manifestations of the human body when balance function is impaired include: low muscle strength and endurance; decreased flexibility of joints and soft tissue flexibility; dysfunction of the central nervous system; decreased visual, vestibular function, and proprioceptive efficiency; reduced tactile input and sensitivity; and reduced spatial perception (You and Wen, 2014)In the field of clinical research, balance testing and training tools have been recognized and accepted by many people as an adjunct to the detection and treatment of patients with balance disorders.

In clinical practice, the most commonly used body composition analysis is measured using the bioelectrical impedance analysis (BIA) method (Zhao and Li, 2016). This method takes advantage of the fact that different components of the human body have different water content and different electrical conductivity, and combines data such as height, weight, gender, and age to estimate the proportion of different components of the human body such as muscle and fat. The differences in the effects of the time factor on BMI, body fat percentage, visceral fat area, skeletal muscle, and Inbody score in this trial were all statistically significant, with reductions in BMI, BFR, and VFA, increases in skeletal muscle, and Inbody score of varying magnitude, and an overall more reasonable body composition, but the results showed that these differences were not related to the exercise intervention factors. The musculoskeletal benefits of exercise are clearly beneficial for middle-aged and older adults (Lim and Goh, 2022; Schaun et al., 2022), and studies have concluded that comprehensive content exercise helps by improving muscle size, muscle strength, balance and physical function in older adults that have decreased because of age (Shahtahmassebi et al., 2022). Due to changing lifestyles and increased academic stress, physical and mental health problems among college students have become increasingly serious, and there are systematic evaluations of qigong exercises considered to improve physical and mental health of college students, with no significant effects on muscle strength found (Lin et al., 2022b). There is also a study of the effects of 8 weeks of resistance training on physical performance and body composition in adolescent athletes, with significant benefits in muscular endurance, strength and static balance (Fayazmilani et al., 2022). These exercises had no statistically significant changes in muscle and effects on balance similar to the present study. It is possible that the age stage or the lifestyle of college students led to a small effect of the intervention in body composition.

Dual-energy X-ray bone densitometry is the most widely used bone densitometry technique at present. In this study, the BMD and BMD Z-value post-test and control group both increased compared with the pre-test, which was statistically significant and may be related to the fact that the BMD of college students is still in a natural growth state at this stage. The interaction of BMD content, BMD Z-value group and time between the test and control groups was not statistically significant, and the differences were not related to the exercise intervention factors. In a 2-year study of healthy young women, exercisers did not have an effect on bone mineral density in the ulnar radius, lumbar spine, and proximal femur after 2 years of physical activity (Mazess and Barden, 1991), which is similar to the results of the present study. Although it is generally accepted that physical activity contributes to bone health and thus reduces fracture risk, a review of the medical literature suggests that, paradoxically, road cycling has a negative impact on bone strength and is therefore a risk factor for proximal femur fractures (Simonovich et al., 2022). However, physical activity is known to have anabolic effects on bone tissue, and it has been shown to increase BMD in young people and adolescents (Courteix et al., 1998). Some studies have shown that the development of body systems is basically mature at the end of puberty and bone mass is in a slow growth period, but the bone density increases under the stimulation of resistance training, indicating that suitable load stimulation at this stage can still promote bone density (Li et al., 2019). Because high-intensity resistance exercise produces a powerful stimulus to bone through muscle pulling contraction, the bone produces adaptive changes after this stimulus, promoting enhanced activity of osteoblasts, making bone formation greater than bone resorption, bone mineral accumulation, and eventually bone mass and bone density both increase. The effect of the exercise intervention in this trial was not statistically significant, which may be related to the design of the exercise intervention content, or to the short duration of the exercise intervention, or because the effect of the exercise intervention was not significant at this stage. The cumulative effect of physical activity on BMD must have a certain amount of time to manifest itself, and many factors must be taken into account in the effect of physical activity on BMD, such as type of exercise, intensity of exercise, frequency of exercise, duration of each exercise, and duration of exercise intervention (Qu, 2020). Studies have shown a correlation between grip strength and free fat mass and bone mineral density (Pelegrini et al., 2022), and physical activity capacity was positively associated with hip BMD in both young women and older men, and although it is unlikely to increase BMD, the findings suggest that maintaining a high level of physical fitness and activity may help prevent bone loss in middle-aged and older adults (Hauger et al., 2020), and 8 weeks of power cycling high-intensity interval training easily improved physical form in overweight female college students and increased femoral bone mineral density in normal weight female college students (Li et al., 2022). Therefore, adolescents need to reduce body fat and safeguard dietary nutrition through scientific and regular exercise to improve overall bone density (Xu et al., 2021). Muscle and bone are anatomically and physiologically connected and together have a regulatory effect on body function. There are few studies on the association between physical function, body composition and bone mineral density in adolescents, and more prospective studies are needed in the future to provide more reference indicators for peak physical function and peak bone mass. The limitations of this study are mainly in the lack of representativeness of the whole group sampling of girls.

## 5 Conclusion

After a comprehensive exercise intervention, the balance ability of female university students was improved significantly. Female college students showed decreases in body composition BMI, body fat percentage, and visceral fat area over time at the university level, with varying increases in skeletal muscle content and Inbody score, and a more reasonable body composition overall; female college students showed increases in bone mineral density content and bone mineral density Z-score over time; these were not related to the comprehensive exercise intervention. The effect of the combined exercise intervention on body composition and bone mineral density in female college students was not statistically significant, which may be related to age and environmental factors in female college students, and may also be related to the type of intervention.

## Data Availability

The original contributions presented in the study are included in the article/Supplementary Material, further inquiries can be directed to the corresponding author.
